# Biomarkers for the Phenotyping and Monitoring of Asthma in Children

**DOI:** 10.1007/s40521-016-0106-0

**Published:** 2016-10-20

**Authors:** Anna James, Gunilla Hedlin

**Affiliations:** 1 Institute of Environmental Medicine, Karolinska Institutet, 17177 Stockholm, Sweden; 2 Department of Women’s and Children’s Health and Centre for Allergy Research, Karolinska Institutet, Astrid Lindgren Children’s Hospital, 17176 Stockholm, Sweden

**Keywords:** Biomarkers, Airway inflammation markers, Allergy diagnosis, Phenotyping asthma, Children

## Abstract

An important issue in relation to the utility and reliability of biomarkers for asthma monitoring is how asthma is defined and characterized. What kind of asthma, or at what stage of the disease is a particular biomarker supposed to add information? Often, the purpose, or usefulness of a biomarker is not made clear. Diagnosis, severity evaluation, and monitoring are all different clinical uses for a biomarker, and confusion may arise when a biomarker is suitable for one of these but not another. When the utility of available biomarkers are discussed, these different roles need to be clarified. Our opinion is that there are four aspects of relevance to asthma, for which biomarkers are required: to diagnose allergies, to evaluate inflammation in the airways, to evaluate hyper-responsiveness, and for certain measures of lung function, such as lung clearance index. These types of biomarkers are needed for the phenotyping and monitoring of asthma. Another important role for biomarkers is, as mentioned above, to monitor asthma in order to follow treatment effects on inflammation and hyper-responsiveness as objective adjuncts to the patients’ own symptom reports and lung function. This review will mainly focus on biomarkers that reflect airway inflammation. In spite of the numerous studies that have been conducted, we still have to remember that the value of biomarkers available for routine use, such as eosinophil counts in blood and sputum and exhaled nitric oxide, have to be interpreted in relation to reported symptoms and lung function. Measures of bronchial hyper-responsiveness, performed either by direct (methacholine challenge) or indirect (exercise or mannitol challenge) methods, could be considered biomarkers but will not be included in this review. On the other hand, diagnosing allergy is not usually useful for monitoring asthma although it is of fundamental importance for the interpretation of most biomarkers that are suitable for monitoring. We have therefore included the different approaches for diagnosing and evaluating allergic sensitization in this review.

## Introduction

Asthma is the single most prevalent handicap among children [1], with a prevalence of up to 15 % reported in certain European countries according to the International Study of Asthma and Allergies in Childhood (ISAAC) [2]. However, this is not one disease with a common underlying mechanism. It is now well known that asthma is a heterogenous disorder with many clinical phenotypes, and the symptoms of airway obstruction and bronchial hyper-responsiveness may be driven by several different biological pathways [3•]. The most recently updated GINA guidelines address these issues by stressing the importance of standardized diagnosis and assessment when considering appropriate treatment strategies [4••]. While the majority of children with asthma have mild or moderate disease and can be adequately controlled by avoiding relevant trigger factors and taking appropriate medications, there is still a group of children with severe asthma in whom symptom control remains poor. A lot of research has been performed in recent years aimed at revealing new biomarkers specific for certain phenotypes, in order to improve the diagnosis and treatment of different asthma subtypes, especially those which are more severe and therapy resistant. New and expensive treatment possibilities require a more individual biomarker approach to target the patients that will benefit the most.

This review will describe biomarkers currently in use for the phenotyping and monitoring of asthma in children but also discuss potential new biomarkers along with their advantages and disadvantages, with a focus on suitability and ease of use in pediatric care.

## Exhaled breath

As a matrix, exhaled air is the most non-invasive kind available, and a range of different methodologies, new and more established, make use of this matrix for the assessment of airway inflammation.

### Exhaled nitric oxide

The most studied is the measurement of exhaled NO (FeNO), which can be analyzed in a quick and easy way, mostly in school-age children although techniques are available to enable measurement even in the very young [5]. FeNO has been shown to correlate with bronchial hyper-responsiveness, blood eosinophils, serum eosinophil cationic protein (ECP), and atopic status/immunoglobulin E (IgE) levels in children [6]. As its levels tend to be reduced by corticosteroid treatment [7], high NO levels may identify children with atopic asthma that are likely to respond to corticosteroid treatment [8]. Furthermore, low exhaled NO in children with asthma may reflect a sub-type of asthma that is less likely to respond to steroid treatment. The exact cutoff values have been described in ATS guidelines published in 2011 [9•].

Nevertheless, despite this vast body of promising findings and even clinical guidelines, the clinical circumstances in which FeNO is able to give the most beneficial results is currently debated, and the link between FeNO and disease severity may be complex [9•]. Petsky and colleagues concluded that measuring FeNO did not improve asthma outcomes in a meta-analysis of three studies examining the benefit of FeNO in guiding the treatment of asthmatic children [10]. The same conclusion is expressed in a recently published review [11••] although the trends toward FeNO measurements contributing to reduced exacerbations and increased medication use are pointed out. Different future uses of FeNO measurements may include analyses of FeNO fluctuation in combination with methods that quantify cross-correlations with symptoms for indicating exacerbation risk, as opposed to the use of isolated FeNO measurements [12]. FeNO measurements in combination with another biomarker may also provide additional clinical information as compared to FeNO alone. Konradsen et al. recently demonstrated that FeNO in combination with blood eosinophil numbers, one local and one systemic marker of Th2-mediated inflammation, were able to identify children with a high asthma morbidity to a greater extent than either marker alone [13]. In another Swedish study of children and young adults, the combination of increased FeNO levels and blood eosinophil counts related to a higher prevalence of severe BHR and uncontrolled asthma [14]. A further interesting example is a study in which regular use of the Asthma Control Test (ACT) to guide therapy was compared to standard visits with or without measurement of FeNO. The results show that both approaches have their advantages and limitations [15•].

### Volatile organic compounds

A more novel approach is that of the analysis of volatile organic compounds (VOC) in exhaled air, or the eNose. This is an “omics” approach in that it involves the profiling of multiple metabolic compounds originating from the lungs and upper airways. The technique used to measure these compounds is usually gas chromatography coupled with mass spectrometry (GC-MS), and this method has been found to differentiate between adults with asthma, COPD, and healthy controls [16, 17]. In children with asthma, VOC analysis enabled prediction of subsequent exacerbations [18] and interestingly, young pre-school children with rhinovirus-induced wheeze were found to have altered exhaled biomarkers both during symptoms and after resolution, possibly reflecting the altered molecular profile which underlies an increased risk of developing asthma [19]. Breath analysis by eNose has even been found to predict response to steroids in patients with asthma more accurately than sputum eosinophils or FeNO [20]. Taken together, these findings suggest that the eNose may be a promising, non-invasive tool worth considering for the future diagnosis and monitoring of children with asthma in highly specialized centers.

### Exhaled breath condensate

Another matrix related to exhaled air is that of exhaled breath condensate (EBC), which is composed of the droplets collected when exhaled breath is cooled. This matrix may be of use for certain future biomarker analyses, such as measures of oxidative stress. Elevated levels of hydrogen peroxide [21], nitrates and nitrites [22], and 8-isoprostane [23] have all been reported in the EBC of patients with asthma compared to healthy controls. However, there are many methodological concerns surrounding the use of EBC. For example, while differences in leukotrienes have been reported in the EBC from asthmatic children compared to healthy controls [24], it has also been shown that any LTs present in EBC are entirely due to salivary contamination [25]. Therefore, the use of EBC for biomarker analysis requires further methodological validation.

## Blood

Obtaining blood samples may be associated with some discomfort in children, but this technique is still considered to be minimally invasive. The blood volumes necessary for cell counting and most biomarker measurements are low and considered to be of low risk for children [26].

### Blood cell counts

Performing differential blood cells counts may provide information regarding asthma phenotypes in adults with asthma [27]. In adults, high blood eosinophil numbers show associations with higher total IgE, lower FEV_1_, more exacerbations, and corticosteroid sensitivity [27, 28]. High blood neutrophil numbers on the other hand tend to correlate with current smoking and non-atopic asthma [27].

Increased blood eosinophils are often observed in pediatric asthma and do show a relationship with disease severity [29]. Another potential for the utility of eosinophil counts in blood is as a marker of response to corticosteroids in pre-school children [30]. However, it has also been shown that blood eosinophil levels do not correlate well with airway eosinophils in children with severe asthma [31•], so use as a single surrogate marker for tissue eosinophila is not recommended. As mentioned above, a combination of eosinophil count and FeNO could reflect both local and peripheral signs of airway inflammation [13, 32]. This combination needs to be evaluated in larger prospective trials for monitoring asthma.

Blood eosinophil counts have also been mentioned in relation to response to omalizumab treatment. Busse and coworkers reported high eosinophil counts to be a potential biomarker capable of reflecting successful omalizumab treatment effects [33]. Further, a combination of three markers, namely, blood eosinophils, FeNO, and periostin, were shown to identify a group of children and adults that had the lowest exacerbation frequency during 48 weeks of omalizumab therapy [34].

Less is known concerning the relevance of blood neutrophil numbers in children, but certain studies do report increased blood neutrophils in children with more severe asthma or in the very young with wheeze [35, 36].

### Immunoglobulin

It is of little doubt that measurement of serum IgE levels can provide important information for the clinical diagnosis of atopic asthma. Measuring allergen-specific IgE levels sheds light on sensitizing allergens, thus enabling the avoidance of trigger factors, and total IgE levels can indicate a general predisposition toward atopic asthma [36]. To highlight the importance of IgE as a biomarker, a specialist NIH task force states that a semi-quantitative screen of specific IgE against common aeroallergens (Phadiatop), in combination with the fx5 panel of food allergens in children younger than 15 years, should be considered core biomarker measurements to be performed in prospective clinical trials and observational studies [26]. Interestingly, more sputum eosinophils have been reported in children with food allergies and asthma, compared to children without food allergies suggesting that there may be a link between food allergy and certain asthma subtypes that requires further investigation [37]. A measurement of total IgE is required before omalizumab treatment can be started but has no role in evaluating the effect of therapy. The basophil allergen threshold sensitivity may be a possible tool in evaluating response to anti-IgE therapy [38].

Certain limitations regarding the methods of total and specific IgE measurements mentioned are that they only provide information about the possible source of the sensitizing allergen, but no information regarding sensitization toward specific allergenic components and thereby knowledge of potential cross-reactivities. Secondly, allergen-specific IgE antibody counts do not always reflect the response of the relevant effector cells and the clinical symptoms that follow upon exposure to allergen. Allergen provocation tests are therefore required, but they cannot always be performed in children with severe asthma due to the risk involved. We therefore describe two approaches which may help to overcome these limitations.

### IgE against allergen components

Cross-sensitization may be explained by the fact that similar epitopes arise in allergens from different sources [39]. Whereas some allergens may be exclusive to a particular source, others may arise from different sources, yet share the same structure and function. New techniques that may help us to identify these allergens involve component specific methodologies, which can determine both the sources of the sensitizing allergens and the origin of the cross-reactive allergens [40]. In addition, it may be possible to predict whether the ensuing reaction experienced by a patient will be mild or severe, based on the allergen to which they are sensitized. Severely asthmatic children with peanut allergy who are allergic to peanut and sensitized to Ara h 1, Ara h 2, and Ara h 3 allergens are at risk of more life-threatening reactions upon exposure to peanut, compared with children sensitized to Ara h 8 [41], a homolog of the main Birch pollen allergen, Bet v 1.

In children with severe asthma, it may be of added benefit to perform such an analysis as Nordlund et al. discovered increased sensitization toward the animal-derived components lipocalin, secretoglobin, and kallikrein in children with problematic, severe asthma as compared to children with more well-controlled asthma [42]. The utility of component resolved analysis in relation to animal dander allergy and asthma is discussed in a review [43•].

### Basophil allergen threshold sensitivity (CD-sens)

A relatively new development that may enable improved assessment of the degree of response to a given allergen is that of the basophil allergen threshold sensitivity test (or CD-sens). CD-sens is alternative to clinical allergen provocation tests performed in vitro which has been shown to correlate with the results of in vivo allergen challenges [44]. This method involves the detection of CD63 on basophils following an in vitro allergen titration, and it can reveal the sensitivity of basophils to a specific allergen. Children with mild and severe asthma who were allergic to cats showed differences in CD-sens despite similar levels of circulating cat-specific IgE [45]. As mentioned above, CD-sens is also a possible biomarker of response to treatment with omalizumab [38].

### Periostin

As its name suggests, periostin was first discovered to be secreted by cells of the periosteum, and it was found to be involved in bone growth and repair [46]. In addition, this matricellular protein is now known to be secreted by many other, predominantly structural cell types throughout the body such as epithelial cells and fibroblasts, and its role seems to be a general involvement in fibrosis and maintenance of the extracellular matrix [47]. Periostin is thought to be involved in type 2-mediated airway inflammation due to its over-expression in epithelial cells from adult asthmatic patients, specific up-regulation by classic type 2 cytokines IL-4 and IL-13, and its ability to determine the response of asthma patients toward anti-IL-13 therapy [48–50].

Despite the interest in periostin as a potential biomarker of type 2 inflammation in asthma, studies examining relationships between circulating periostin levels and airway eosinophilia, as well as other type 2 biomarkers, are relatively few, mostly limited to adults, and some have shown conflicting results [51, 52]. Nevertheless, the majority of published reports in adults do demonstrate modest, yet significant relationships between circulating periostin with other proposed markers of type 2 inflammation, including exhaled NO, sputum, and blood eosinophils or total IgE [53–55].

However, despite the initial associations with eosinophilic asthma observed in adults, periostin is unlikely to be a useful biomarker of type 2 inflammation in children. Small increases in periostin levels have been reported in children with asthma compared to healthy controls at certain, but not all, ages [56], and moderate relationships with blood eosinophils and IgE have also been observed [57]. However, baseline periostin levels are high in children, most likely due to bone growth, which may mask changes due to local release within the airways. This is a major confounding factor and most likely the reason for inconsistent findings regarding periostin as a marker of type 2 asthma in children. No correlations between serum periostin and FeNO, blood eosinophils, or IgE were found in school-age children with moderate or severe asthma [13], or in children aged 6 to 11 with various manifestations of allergic disease, including asthma [58].

### YKL-40

One unmet need in biomarker research is the discovery of markers which may reflect non-type 2 driven asthma. A mediator which has shown potential for this purpose is YKL-40, a chitinase-like protein which is elevated in the serum of both school-age children and adults with severe asthma. Increased circulating YKL-40 consistently associates with reduced lung function, strengthening a possible relationship with disease severity [59–62]. The exact biological function of YKL-40 remains unclear, but the fact that it associates with measures of airway remodeling such as bronchial wall thickness and subepithelial fibrosis [59, 60] and increases the proliferation of bronchial smooth muscle cells [63, 64] is in line with an involvement in airway remodeling and fibrotic lung disease [65].

YKL-40 levels are higher in adults with COPD compared to asthma [62] and, preliminarily, in children with BPD compared to asthma [66], suggesting that the chitinases are not Th2-specific markers of airway disease. Also, correlations often exist between YKL-40 levels and neutrophilic, rather than eosinophilic inflammation [60, 67]. However, further studies are required to establish whether YKL-40 measurements are of clinical value and if YKL-40 has any potential in the monitoring of persistent asthma.

### Others

Novel blood biomarkers that may be associated with asthma in children are continuously emerging, and it is beyond the scope of this review to describe them all, but thymic stromal lymphopoetin (TSLP) is an epithelial-derived “alarmin” cytokine worth mentioning as a promising example [68]. Little is known about its circulating levels in pediatric cohorts, although an increase in children with asthma compared to controls, as well as an association with asthma control, has been reported [69]. Measuring circulating 25-OH Vitamin D levels in the clinic may also be of interest as several pediatric studies now describe an association between Vitamin D deficiency and increased risk of asthma, as well as asthma severity [70–72]. The field of biomarker discovery is exploding with novel biomarkers due to the increasing application of “omics” technologies. For example, Hamsten and colleagues recently drew attention to the differential expression of chemokine ligand 5 (CCL5), hematopoietic prostaglandin D synthase (HPGDS), and neuropeptide S receptor 1 (NPSR1) in the plasma of children with asthma using an antibody-based proteomic array [73]. Further validation will reveal whether these new “hits” are of clinical value.

## Lower airway samples

### Sputum

Induced sputum is a relatively safe, semi-invasive method which can be generally be performed well by school-age children assisted by specially trained staff [74]. Different cellular compositions have been described in children (eosinophilic, neutophilic, mixed granulocytic, and paucigranulocytic), which may relate to different asthma phenotypes [75]. It has been shown that sputum eosinophil numbers are higher in atopic as compared to non-atopic childhood asthma [76]. As well as cellular profile, it is also possible to measure soluble inflammatory mediators in sputum supernatants. Cytokine profiles in sputum supernatant from children with asthma have both been described as altered [76], and unaltered as compared to healthy controls [77]. Interestingly, recent findings from our own group suggest that sputum IL-26 may be a novel biomarker of pediatric asthma without signs of Th2-mediated or eosinophilic inflammation [78]. This is of particular interest as the biological mechanisms underlying non-Th2-driven asthma remain elusive. However, it must be noted that there is no evidence to suggest that the analysis of induced sputum samples is clinically useful for the monitoring of asthma in children. Sputum cell profiles have been shown to be unstable over time [75] and have failed to successfully guide anti-inflammatory treatment [79].

### Bronchoscopic procedures

Obtaining bronchial brushings, biopsies, and lavage fluid from children is possible, but these are invasive procedures that can only be performed in specialized centers and tend only to be performed to evaluate airway inflammation in children with very severe therapy-resistant asthma [80]. Although not routinely performed in children, valuable research findings have been obtained in recent years from the transcriptome analyses performed using bronchial brushings (epithelial cells) collected from adults. Woodruff and colleagues established a “Th2-high” signature by grouping subjects based on elevated expression of three bronchial epithelial genes: CLCA1, periostin, and serpinB2. Patients in this Th2-high group had more airway eosinophils and bronchial hyper-responsiveness and were responsive to inhaled corticosteroids therapy as opposed to those in the “Th2-low group” [49].

## Urine

Collecting urine is an extremely non-invasive process which would make it an ideal matrix for the measurement of biomarkers and monitoring of childhood asthma. However, there are currently no urinary biomarkers that are routinely examined in a clinical setting to assess airway inflammation in children with asthma. Nevertheless, promising findings from research studies suggest that there may be a place for routine urine sampling in children with asthma, particularly for the measurement of eicosanoids and their metabolites.

### Eicosanoids

The involvement of leukotrienes, prostaglandins, and other arachidonic acid-derived lipid mediators in asthma and airway inflammation has long been known. The cysteinyl leukotrienes (Cys-LTs) are among the most potent bronchoconstrictive agents known and also have chemotactic, mucous secretory, and pro-fibrotic effects in the airways [81]. Different prostaglandins (PGs) acting at their respective receptors are also clearly involved in both pro- (e.g., PGD_2_) and anti-inflammatory (e.g., PGE_2_) processes in airway disease [82]. Despite their well-known biological activity, these mediators have sometimes proven difficult to measure due to their rapid metabolism and clearance from the circulation which has hampered their use as biomarkers [83, 84]. However, as an alternative to blood, the analysis of lipid mediator metabolites (rather than primary compounds) excreted into the urine has emerged as a non-invasive biomarker. Reproducible liquid chromatography coupled to mass spectrometry (LC-MS)-based platforms have recently been developed specifically for this purpose [85]. Two urinary eicosanoid metabolites which are considered to be of particular interest in asthma are 11β-PGF2α and LTE_4_ by virtue of their abilities to reflect mast cell and eosinophil activation, respectively.

Urinary LTE_4_ has been found to increase during asthma exacerbations, caused by both inhaled aspirin and allergen challenges [86–88]. Furthermore, drugs that block Cys-LT synthesis significantly decrease urinary LTE_4_ levels [89], whereas corticosteroids do not affect urinary LTE_4_ [86]. Primary PGD_2_ is not detectable in urine, and its earliest appearing metabolite is 11β-PGF2α [90]. Several lines of evidence suggest that 11β-PGF2α levels in the urine reflect mast cell activation in the lung. Its levels are increased by inhaled allergen challenge and inhaled mannitol challenge, as well as during aspirin- and exercise-induced bronchoconstriction in susceptible individuals [91]. Again, mast cell PGD_2_ release is not significantly affected by corticosteroid treatment [92], although the mast cell-stabilizing drug sodium cromoglycate may prevent its release [93].

Preliminary findings from our group suggest that children with asthma who had high levels of urinary LTE_4_ or 11β-PGF2α (above the 75th percentile), as compared to those with low levels (below the 25th percentile), had a lower FEV_1_, increased bronchial responsiveness, increased IgE, and increased blood eosinophils, factors which may well reflect a more type 2-driven airway inflammation [94]. The future of urinary lipid metabolites as phenotype-specific biomarkers in asthma is likely to develop further as the platforms used evolve to incorporate increased numbers of metabolites, thus enabling a more complete overview of arachidonic acid metabolism [85].

### Eosinophil-derived neurotoxin

Another class of mediators that may be measured in the urine, and which have received renewed interest in recent years, are eosinophil-derived proteins such as eosinophil protein X (EPX, also currently known as EDN, eosinophil-derived neurotoxin). These are eosinophil granule-derived proteins, the advantage of which being that they may reflect eosinophil activation, rather than eosinophil numbers, which may be of more relevance to active inflammatory disease processes. Over the years, research studies have shown conflicting findings, but a recent meta-analysis taking into account 27 childhood studies conducted over a 20-year period concluded that over 70 % of these did show an association between EPX and childhood asthma, thus implicating a clinical role for EPX measurements [95]. Future studies will confirm whether this is the case and if EDN in urine could be used for monitoring of asthma in children.

## Conclusions

Despite the continuing emergence of promising new biomarkers for the phenotyping and monitoring of asthma in children, we still must acknowledge that our understanding of the molecular mechanisms underlying different asthma phenotypes is limited. Of all the biomarkers discussed above, only one or two are currently used in the clinic, and these mostly in specialist clinics. The most studied biomarker is FeNO, yet in spite of this, it is still considered equivocal although there is evidence that high levels can predict risk of exacerbation. Generally, biomarkers or physiological measures that precisely and conclusively define sub-phenotypes of asthma are lacking. Before many of the potential biomarkers described in this review can enter clinical practice, a great deal more validation is required in studies of well-characterized asthmatic children [96•]. Normal ranges need to be established, and stability over time must be examined in longitudinal studies. We need to know more about the effects of medications on biomarker measurements, especially for biomarkers which are proposed to guide different treatments [97••]. Knowledge of confounding factors is vital as highlighted above by the effect of age, and thereby growth, on periostin levels. Finally, and most importantly, any new biomarkers need to be easily sampled and easy to interpret at the point of care in order to provide better treatment for those children who need it the most. A combination of non-invasive biomarkers and the use of electronic devices to enable patients to report symptoms and lung function will hopefully initiate a more personalized approach to health care and improve adherence to therapy.
